# Diversification of *Escherichia albertii* H-Antigens and Development of H-Genotyping PCR

**DOI:** 10.3389/fmicb.2021.737979

**Published:** 2021-11-01

**Authors:** Koji Nakae, Tadasuke Ooka, Koichi Murakami, Yukiko Hara-Kudo, Naoko Imuta, Yasuhiro Gotoh, Yoshitoshi Ogura, Tetsuya Hayashi, Yasuhiro Okamoto, Junichiro Nishi

**Affiliations:** ^1^Department of Pediatrics, Kagoshima University Hospital, Kagoshima, Japan; ^2^Department of Microbiology, Kagoshima University Graduate School of Medical and Dental Sciences, Kagoshima, Japan; ^3^Center for Emergency Preparedness and Response, National Institute of Infectious Diseases, Tokyo, Japan; ^4^National Institute of Health Sciences, Kawasaki, Japan; ^5^Department of Bacteriology, Faculty of Medical Sciences, Kyushu University, Fukuoka, Japan; ^6^Department of Infectious Medicine, Division of Microbiology, Kurume University School of Medicine, Kurume, Japan

**Keywords:** *Escherichia albertii*, flagellin, *fliC*, H-antigen, genotyping

## Abstract

*Escherichia albertii* is a recently recognized human enteropathogen that is closely related to *Escherichia coli*. As *E. albertii* sometimes causes outbreaks of gastroenteritis, rapid strain typing systems, such as the O- and H-serotyping systems widely used for *E. coli*, will be useful for outbreak investigation and surveillance. Although an O-genotyping system has recently been developed, the diversity of *E. albertii* H-antigens (flagellins) encoded by *fliC* genes remains to be systematically investigated, and no H-serotyping or genotyping system is currently available. Here, we analyzed the *fliC* genes of 243 genome-sequenced *E. albertii* strains and identified 73 sequence types, which were grouped into four clearly distinguishable types designated *E. albertii* H-genotypes 1–4 (EAHg1–EAHg4). Although there was a clear sign of intraspecies transfer of *fliC* genes in *E. albertii*, none of the four *E. albertii* H-genotypes (EAHgs) were closely related to any of the 53 known *E. coli* H-antigens, indicating the absence or rare occurrence of interspecies transfer of *fliC* genes between the two species. Although the analysis of more *E. albertii* strains will be required to confirm the low level of variation in their *fliC* genes, this finding suggests that *E. albertii* may exist in limited natural hosts or environments and/or that the flagella of *E. albertii* may function in a limited stage(s) in their life cycle. Based on the *fliC* sequences of the four EAHgs, we developed a multiplex PCR-based H-genotyping system for *E. albertii* (EAH-genotyping PCR), which will be useful for epidemiological studies of *E. albertii* infections.

## Introduction

*Escherichia albertii* is a recently recognized human enteropathogen and an avian pathogen (Albert et al., 1992; Huys et al., 2003; Oaks et al., 2010; Ooka et al., 2012). *E. albertii* is often misidentified as enteropathogenic *Escherichia coli* (EPEC) or enterohemorrhagic *E. coli* (EHEC) due to similar phenotypic and genetic characteristics, including similar biochemical properties and possession of a locus of enterocyte effacement (LEE) encoding a type III secretion system (Ooka et al., 2012; Gomes et al., 2020). In addition, as multiple outbreaks of *E. albertii* have recently been reported (Konno et al., 2012; Ooka et al., 2013; Masuda et al., 2020), rapid strain typing systems, such as the PCR-based O- and H-genotyping systems widely used for *E. coli* (Iguchi et al., 2015; Banjo et al., 2018), should be useful for *E. albertii* outbreak investigation and surveillance. In *E. albertii*, although only a genotyping system based on the variation in O-antigen biosynthesis genes has been developed thus far (Ooka et al., 2019), rapid, and low-cost effective H-genotyping system are useful to increase the discrimination power and to assist epidemiological studies in combination with O-genotyping system.

H-antigens (flagellins) are used for the serotyping of strains in many Gram-negative bacteria (Orskov and Orskov, 1992). In *E. coli*, flagellin is encoded by the *fliC* gene in the *fliY*-*T* region on the chromosome or its homologs, such as *fliK*, *fliA*, and *fimA* (Ratiner, 1998; Wang et al., 2003; Tominaga, 2004; Feng et al., 2008; Ratiner et al., 2010), and a total of 53 H-antigens have been identified thus far. The flagellar filament is composed of a single protein, flagellin. Flagellin is composed of four major domains: the N- and C-terminal domains (D0 and D1, respectively) form the inner and outer tubules of the flagellum, respectively, and internal D2 and D3 domains are exposed on the surface of the flagellar filament. The D0 and D1 domains are highly conserved among bacterial species, whereas the D2 and D3 domains are highly variable even between strains belonging to the same species (Samatey et al., 2001). In *E. albertii*, the gene cluster associated with flagellar biosynthesis and its regulation, including the *fliY-T* region, is conserved in most strains (Ooka et al., 2015). Although the flagellum is not produced under routine culture conditions, it has recently been revealed that its production is induced under conditions of low temperature and nutrient limitation (Ikeda et al., 2020; Murakami et al., 2020). However, the sequence variation of the *fliC* gene has not yet been examined. To clarify this issue, we systematically analyzed the *fliC* genes of 243 *E. albertii* strains sequenced thus far and compared them with the sequences of 53 known *E. coli* H-antigens. In addition, we attempted to develop a multiplex PCR-based H-genotyping system for *E. albertii* strains based on the sequence diversity of their *fliC* genes.

## Materials and Methods

### *Escherichia albertii* Genome Sequences and Strains Analyzed in This Study

In this study, we analyzed the 243 *E. albertii* genome sequences used in our previous study (Ooka et al., 2019). Detailed information of the strains is shown in Supplementary Table 1. The strain information for the 92 *E. albertii* strains used for the evaluation of EAH-genotyping PCR is provided in Supplementary Table 2.

### Identification of *fliC* Genes

In previously sequenced *E. albertii* genomes, the *fliC* gene has been found to be located between *fliA* and *fliD* (Ooka et al., 2015). In the first-step analysis, as it is known that *fliC* sequences are highly diverse in *E. coli* (Ratiner et al., 2010), the *fliC*-containing regions of the 243 *E. albertii* genomes were identified by blastn search using the *fliA* and *fliD* sequences of *E. albertii* strain CB9786 as queries, with an *E*-value threshold of 0.01. The *fliC* gene of each strain was then manually annotated with *in silico* Molecular Cloning Genomics Edition software version 7.29L (IMC-GE; In Silico Biology, Japan). For the strains not identified the *fliC* gene in the first-step analysis, we performed the second-step analysis by blastn search using the *fliC* sequences identified in the first step analysis or the primer sequences designed for EAH-genotyping PCR, which was described below, as queries, with an *E*-value threshold of 0.01.

### Sequence Comparison and Phylogenetic Analysis

Multiple alignment of nucleotide sequences of the *fliC* gene of *E. albertii* strains and amino acid sequences of the flagellin of *E. albertii* and *Salmonella* Typhimurium strain SJW1103 was prepared using GENETYX (version 15.0.1). After identical sequences showing no SNPs were deduplicated, the nucleotide sequence alignment of *E. albertii fliC* genes with those of 53 known *E. coli* H-serotypes (H1–H56, but missing H13, H22, and H50; Supplementary Table 3) was performed using the ClustalW program in MEGA (version 7.0.26) with the default parameters (Kumar et al., 2016).

The phylogenetic analysis of *fliC* genes was performed with MEGA using the obtained multiple alignment. A phylogenetic tree was reconstructed by the neighbor-joining (NJ) method with the *p*-distance model. Bootstrap analysis with 1000 replicates was performed to assess the significance of internal branching. The core-gene SNP-based maximum-likelihood (ML) phylogenetic tree of the 243 *E. albertii* strains was constructed previously (Ooka et al., 2019) using RAxML v8 (Stamatakis, 2014) and displayed and annotated using iTOL v4^1^ (Letunic and Bork, 2016).

### Development of a Multiplex PCR-Based *Escherichia albertii* H-Genotyping System

Based on the variation in the sequences of the *E. albertii fliC* genes, we designed four pairs of PCR primers to specifically detect each of the four *E. albertii* H-genotypes (EAHgs) (Table 1). As a positive control for PCR and a genetic marker of *E. albertii*, one primer pair targeting an *E. albertii*-specific region (E_al_1_NF/NR primers) (Ooka et al., 2019) was also included in the primer set. Template DNA for PCR was prepared by the alkaline boiling method. KOD -Multi&Epi- DNA polymerase (TOYOBO, Osaka, Japan) was used for PCR. Each reaction mixture (25 μl) contained 1 μl of template DNA, each primer at 1 μM, and 0.5 U of polymerase. PCR was performed with 25 cycles of 94°C for 2 min for initial denaturation, followed by 10 s at 98°C, 30 s at 60°C, and 60 s at 68°C. The PCR products were analyzed by agarose electrophoresis using 2% agarose S (Nippon Gene, Tokyo, Japan).

**TABLE 1:** Primer information for EAH-genotyping PCR.

Primer name	Sequence (5′–3′)	Product size (bp)	Reference strain	Note
EAHg_F (common)	CAGGTTGGCGCGAATGA TGG	–	–	–
EAHg1_R	GCATCTAGTTTAACTGACTG	167	EC05-81	EAHg1
EAHg2_R	GGTTGCAGAAGTAACGGTAG	309	NIAH_Bird 25	EAHg2
EAHg3_R	GGCTGACCAGTTTGTTTCGC	404	NIAH_Bird 3	EAHg3
EAHg4_R	GTACCATTTGTACCAGCAAG	515	NIAH_Bird 5	EAHg4
E_al_1_NF	CAGTCGATGGTTTCACCTGA	731	–	*E. albertii*-specific
E_al_1_NR	ACACCGTGGCGAAATGGCA			

## Results

### Identification and Sequence Comparison of *fliC* Genes in *Escherichia albertii* Genomes

Among the 243 *E. albertii* genomes examined by the first and second step analysis, intact and partial sequence of the *fliC* genes were identified in 231 and 9 genomes and no sequences were detected in the remaining 3 genomes (Supplementary Table 1). Through the clustering analysis of the 231 of intact *fliC* gene sequences, we identified a total of 73 sequence types with one or more SNPs (Supplementary Table 1), among which 42 were singletons, and 31 were composed of sequences from multiple genomes (named clusters C1–C31).

### Phylogenetic Analysis of *Escherichia albertii fliC* Genes With Those of *Escherichia coli* as References

**FIGURE 1:**
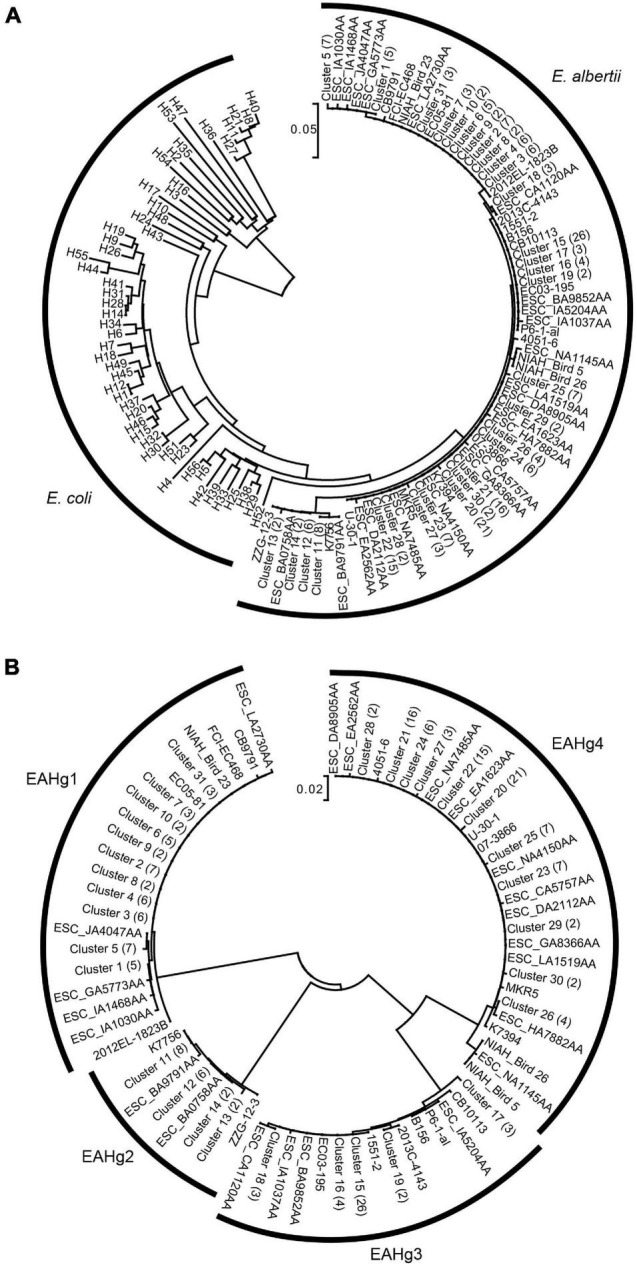
**(A)** Neighbor-joining tree of 73 *fliC* sequences identified from 231 *E. albertii* strains. The sequences of 53 known *fliC* genotypes of *E. coli* are included as references. The number of strains with identical *fliC* sequences is shown in parentheses for each cluster. **(B)** Neighbor-joining tree of only the 73 *fliC* sequences of *E. albertii*. The number of strains with identical *fliC* sequences is shown in parentheses for each cluster.

The phylogenetic analysis of the 73 *fliC* sequences identified in *E. albertii* with 53 *fliC* sequences of known *E. coli* H-serotypes as references revealed that the *fliC* genes of *E. albertii* formed a monophyletic branch, separate from those of *E. coli* (Figure 1A). To obtain more detailed information on the sequence variation in *E. albertii fliC* genes, we performed a phylogenetic analysis of only the *E. albertii fliC* genes (Figure 1B). This analysis revealed that the *E. albertii fliC* genes can be divided into four distinct groups, in which the nucleotide sequence identities between the groups were less than 90%, and those within each group were over 97%. We defined these four groups as the genotypes of *E. albertii fliC* genes and named them *E. albertii* H-genotypes 1–4 (EAHg1–EAHg4). In addition, by the primer screening analysis for the nine strains with partial sequences of the *fliC* gene, all nine strains could be genotyped into either of the four EAHgs. Together with the results of *in silico* analysis of 231 genome-sequenced strains, the most dominant type among the 243 *E. albertii* genomes was EAHg4 (109 strains; 44.9%), followed by EAHg1 (59 strains; 24.3%), EAHg3 (50 strains; 20.6%), EAHg2 (22 strains; 9.0%), and no *fliC* gene (3 strains; 1.2%) (Table 2).

**TABLE 2:** Summary of *in silico*- and PCR-based EAH genotyping.

EAH-genotype	Number of strains (%)	
	*In silico* *	PCR
EAHg1	59 [1] (24.3%)	24 (26.1%)
EAHg2	22 [0] (9.0%)	16 (17.4%)
EAHg3	50 [2] (20.6%)	13 (14.1%)
EAHg4	109 [6] (44.9%)	39 (42.4%)
Not identified	3 (1.2%)	0 (0%)
Total	243	92

*The number of strains genotyped by primer screening were shown in brackets.

Amino acid sequence comparison of the representative *fliC*-encoded flagellin of the four genotypes and *Salmonella* Typhimurium strain SJW1103 revealed that while the sequences of the D0 and D1 domains were highly conserved, those of the D2 and D3 domains were variable (Supplementary Figure 1).

### Development and Evaluation of the Multiplex H-Genotyping PCR System for *Escherichia albertii*

**FIGURE 2:**
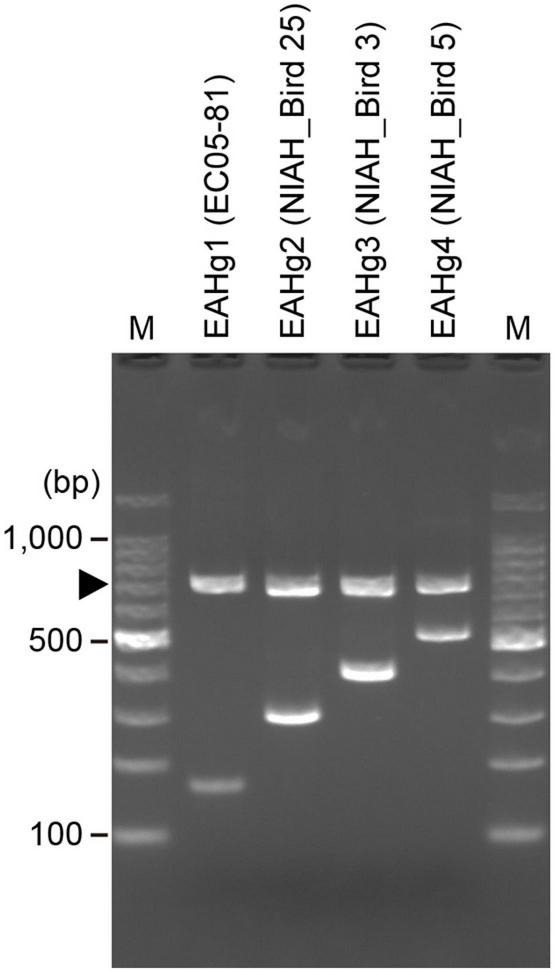
Electrophoresis patterns obtained by EAH-genotyping PCR. A total of four strains representing the four EAHgs were analyzed using a PCR primer mix designed in this study. Strain names are indicated in parentheses. An arrowhead indicates the bands derived from the *E. albertii*-specific primer pair E_al_1_NF/NR. Lane M, 100 bp DNA ladder.

We designed a multiplex PCR primer set (Table 1) based on the sequence variation in the *fliC* genes of the four EAHgs. A forward universal primer was designed based on the highly conserved sequences of all four EAHgs, and reverse primers were designed based on the variable regions of each EAHg (Supplementary Figure 2), so that the amplicons generated with the combinations of the universal forward primer and the reverse primers exhibited a ladder pattern that ranged from 167 to 515 bp. One *E. albertii*-specific primer pair (E_al_1_NF/E_al_1_NR) (Ooka et al., 2015) was included in the primer set as a marker to detect *E. albertii* and as a positive control for PCR. The examination of the primer set in four strains representing each of the four EAHgs confirmed that the primer set yielded PCR products of the expected sizes for each genotype (Figure 2). To validate the PCR-based genotyping system, we performed the comparison of *in silico* and PCR-based genotyping on the same dataset of genomes. In this analysis, only 42 strains were used because the remaining 201 strains are not available in our laboratories. As shown in Supplementary Figure 3, all of the 42 strains exhibited the same results between *in silico* and the PCR-based genotyping. In addition, we applied the mixed DNA samples containing four strains with different EAH-genotypes as a template for the system to evaluate the specificity and obtained the primer set yielded PCR products of the expected sizes for four genotypes (Supplementary Figure 4).

To further evaluate the performance of the system, we determined the H-genotypes of 92 *E. albertii* strains using this system. These strains were isolated from diarrheal patients and birds in various regions of Japan. In this analysis, we were able to genotype all strains (Supplementary Table 2). Similar to the results of *in silico* analysis of the 240 genome-sequenced strains, EAHg4 (48 strains; 43.6%) was found to be predominant, followed by EAHg1 (29 strains; 26.4%), EAHg2 (18 strains; 16.4%), and EAHg3 (15 strains; 13.6%) (Table 2). These results suggest that our system is useful for the H-genotyping of *E. albertii* and that the four genotypes cover the most of the diversity of H-genotypes in the *E. albertii* lineage.

### Distribution of the 4 *Escherichia albertii* H-Genotypes and the 40 *Escherichia albertii* O-Genotypes in Genome-Sequenced *Escherichia albertii* Strains

**FIGURE 3:**
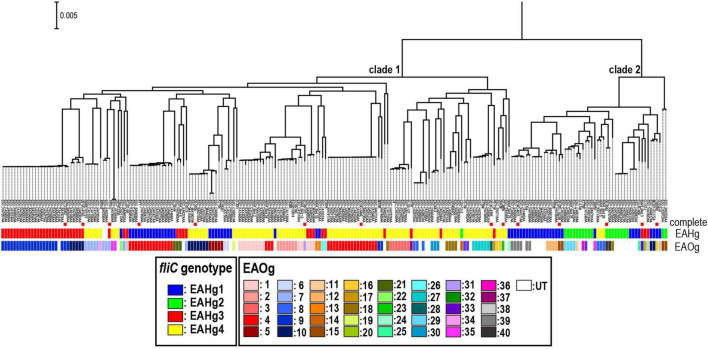
Phylogenetic view of the 225 *E. albertii* strains that have been genome sequenced thus far (9) and the distribution of the four EAHgs and the 40 EAOgs in these strains. Strain names are indicated at each tip, and the 14 completely sequenced strains are indicated. Information on the distribution of the four EAHgs and the 40 EAOgs are also shown.

Finally, we investigated the relationship between the phylogeny of the genome-sequenced strains used in this study and the distribution of the four EAHgs and the 40 Escherichia albertii O-genotypes (EAOgs), identified in our previous study (Ooka et al., 2019), in these strains by mapping H- and O-genotype information in a core-gene sequence-based ML phylogenetic tree of the strains (Figure 3). This analysis revealed that although very closely related strains shared the same H-genotype, each EAHg appeared in multiple sublineages in both clades 1 and 2, suggesting relatively frequent within-species transfer of *fliC* genes in *E. albertii*. In addition, there is no correlation between the combination of the H- and O-genotypes and their phylogenetic relationship.

## Discussion

In this study, we analyzed the sequence variation of *fliC* genes among 231 genome-sequenced *E. albertii* strains and identified 73 sequence types, which were grouped into four clearly distinguishable genotypes (EAHg1–EAHg4). The four genotypes showed >97% sequence identity within each group and <90% identity between groups. As seen in *E. coli fliC* genes (Samatey et al., 2001), the sequences encoding domains D0 and D1 (corresponding to the N- and C-terminal regions of flagellin, respectively) were highly conserved, but those of domains D2 and D3 (forming the surface exposed region) were variable between H-genotypes (Supplementary Figure 1). In our previous analysis (Ooka et al., 2015), we revealed that flagellar biosynthesis-related genes other than *fliC* show high conservation of nucleotide sequences (>90% identity). Therefore, it appears that the *fliC* gene is under a certain amount of immunological selection in its hosts or environmental selection. However, it was notable that only four H-genotypes were identified in *E. albertii*, in sharp contrast to the situation in *E. coli*, in which as many as 53 H-genotypes have been identified. Although the analysis of more *E. albertii* strains will be required to confirm the low level of variation in their *fliC* genes, this finding may suggest the possibility that this species is living in limited natural hosts or environments and/or that their flagella are required in a limited stage in their life cycle. This possibility may receive some support from the recent finding that flagella are produced only at lower temperatures and under nutrient-limited conditions (Ikeda et al., 2020; Murakami et al., 2020). It is also noteworthy that although a low level of sequence variation was observed for *E. albertii fliC* genes, we detected a sign of relatively frequent within-species transfer of this gene in *E. albertii* (Figure 3). However, none of the four H-genotypes identified in this study were closely related to any of the 53 H-genotypes of *E. coli*, suggesting the absence or very rare occurrence of interspecies transfer of *fliC* between *E. albertii* and *E. coli*. This is in marked contrast to the situation for O-antigen biosynthesis loci, which show clear signs of interspecies transfer between the two species (Ooka et al., 2019). Although the mechanism(s) generating such a difference is currently unknown, there may be some restriction of the flagellin sequence of *E. albertii*.

Although only four H-genotypes were identified in *E. albertii*, the multiplex PCR system that we constructed to identify H-genotypes in *E. albertii* will be a useful tool for epidemiological studies of *E. albertii* infections, particularly if used in combination with the O-genotyping system that we previously constructed (Ooka et al., 2019). In addition, the H-genotyping system will be useful for further analyzing the diversity of *fliC* genes in *E. albertii* or searching for additional genotypes.

## Data Availability Statement

The original contributions presented in the study are included in the article/Supplementary Material, further inquiries can be directed to the corresponding author.

## Author Contributions

KN and TO designed the study and wrote the manuscript. KM and YH-K provided the samples. KN, TO, YG, NI, and YoO analyzed the data. TO, YaO, TH, and JN were responsible for supervision and management of the study. All authors contributed to the article and approved the submitted version.

## Conflict of Interest

The authors declare that the research was conducted in the absence of any commercial or financial relationships that could be construed as a potential conflict of interest.

## Publisher’s Note

All claims expressed in this article are solely those of the authors and do not necessarily represent those of their affiliated organizations, or those of the publisher, the editors and the reviewers. Any product that may be evaluated in this article, or claim that may be made by its manufacturer, is not guaranteed or endorsed by the publisher.

## Footnotes

1
https://itol.embl.de
