# Supplementary material for: Diversification of Escherichia albertii H-Antigens and Development of H-Genotyping PCR
Source: Front Microbiol. 2021 Nov 1;12:737979. doi: 10.3389/fmicb.2021.737979 (PMC8591213; doi:10.3389/fmicb.2021.737979)
Supplement: Supplementary file 1 [file Data_Sheet_1.PDF]

Supplementary Figure 1

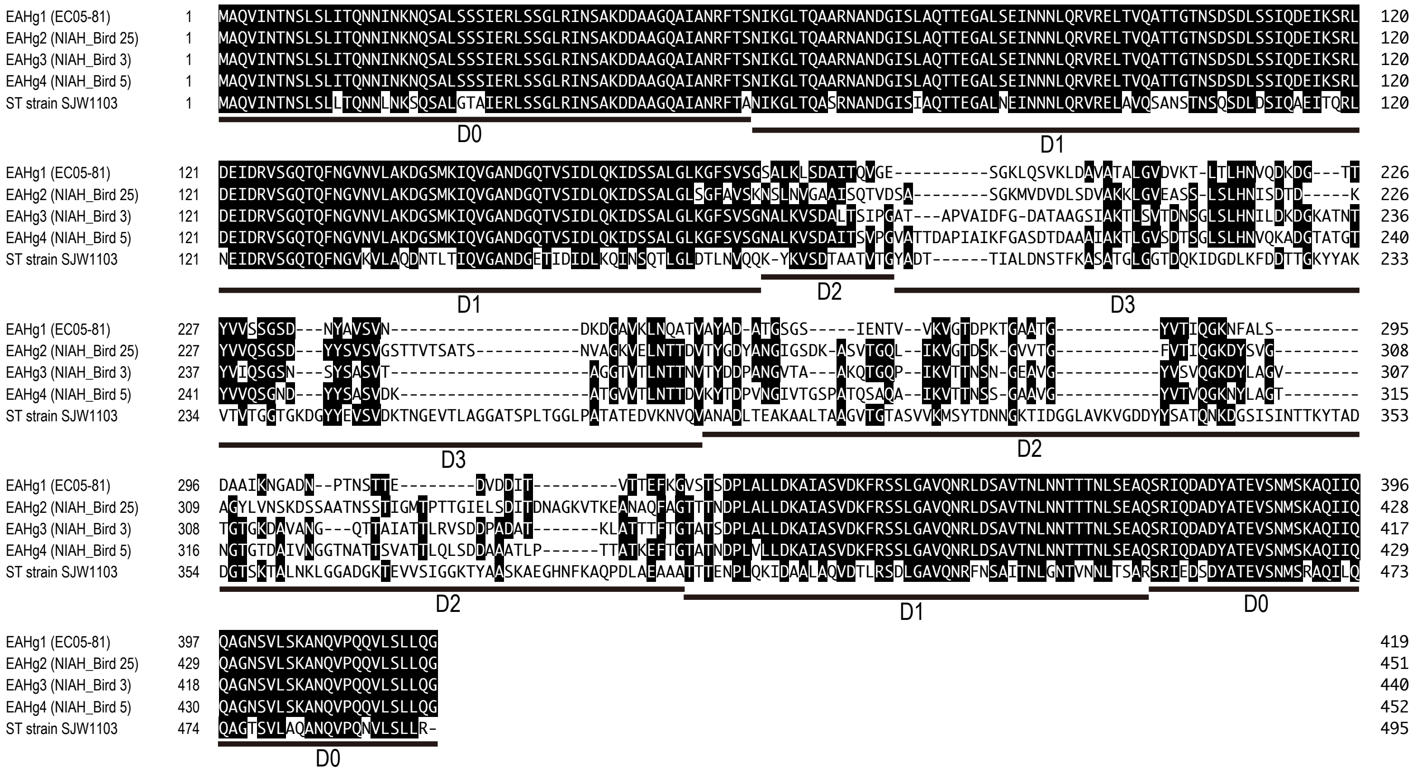

Supplementary Figure 1. Amino acid sequence comparison of representative flagellins of the four EAH-genotypes and *Salmonella* Typhimurium (ST) strain SJW1103. Strain name of each EAH-genotype are shown in parentheses. The assignment of domains (D0-D3) was performed based on the flagellin of ST strain SJW1103.

Supplementary Figure 2

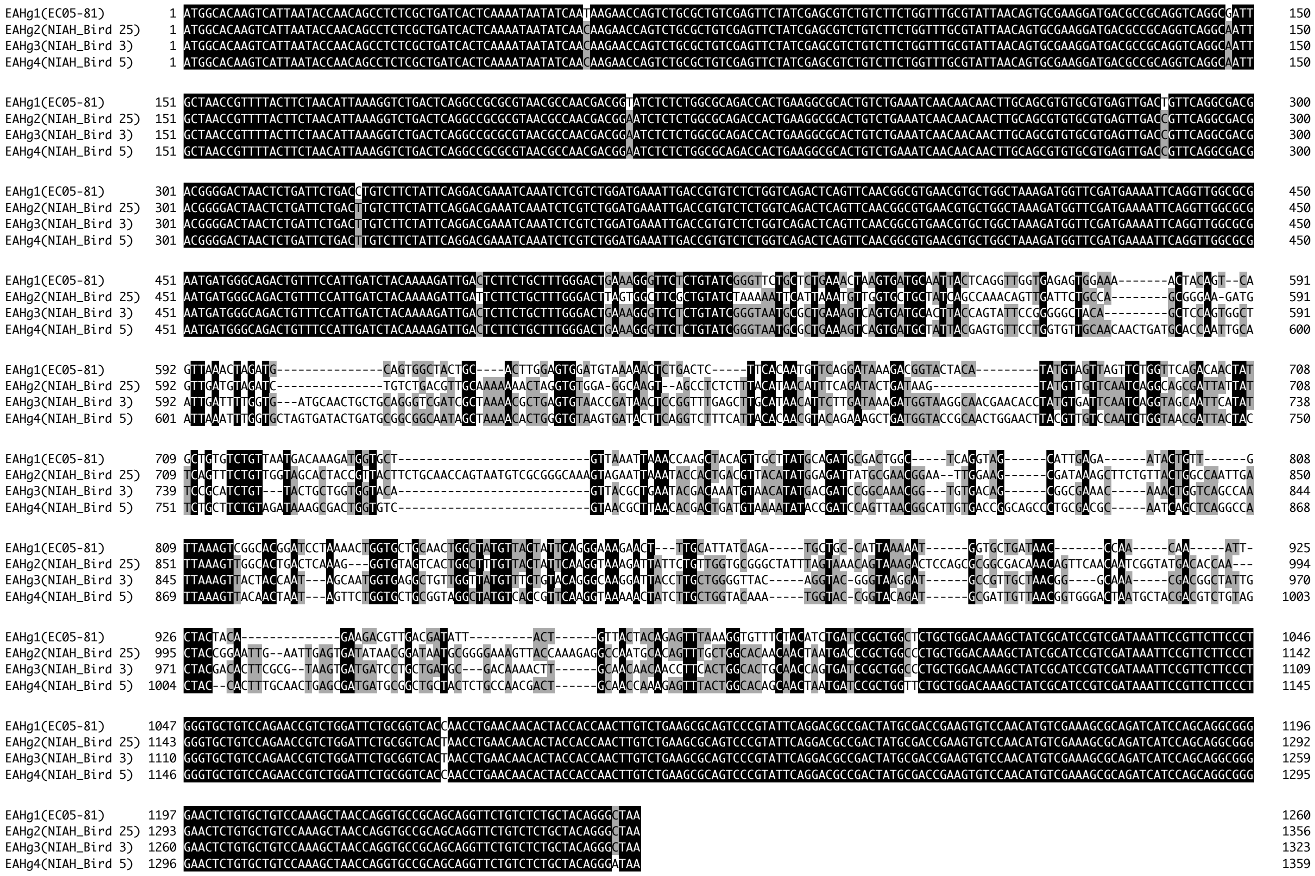

Supplementary Figure 2. Nucleotide sequence comparison of representative *fliC* alleles of the four EAH-genotypes. Strain names are shown in parentheses.

Supplementary Figure 3

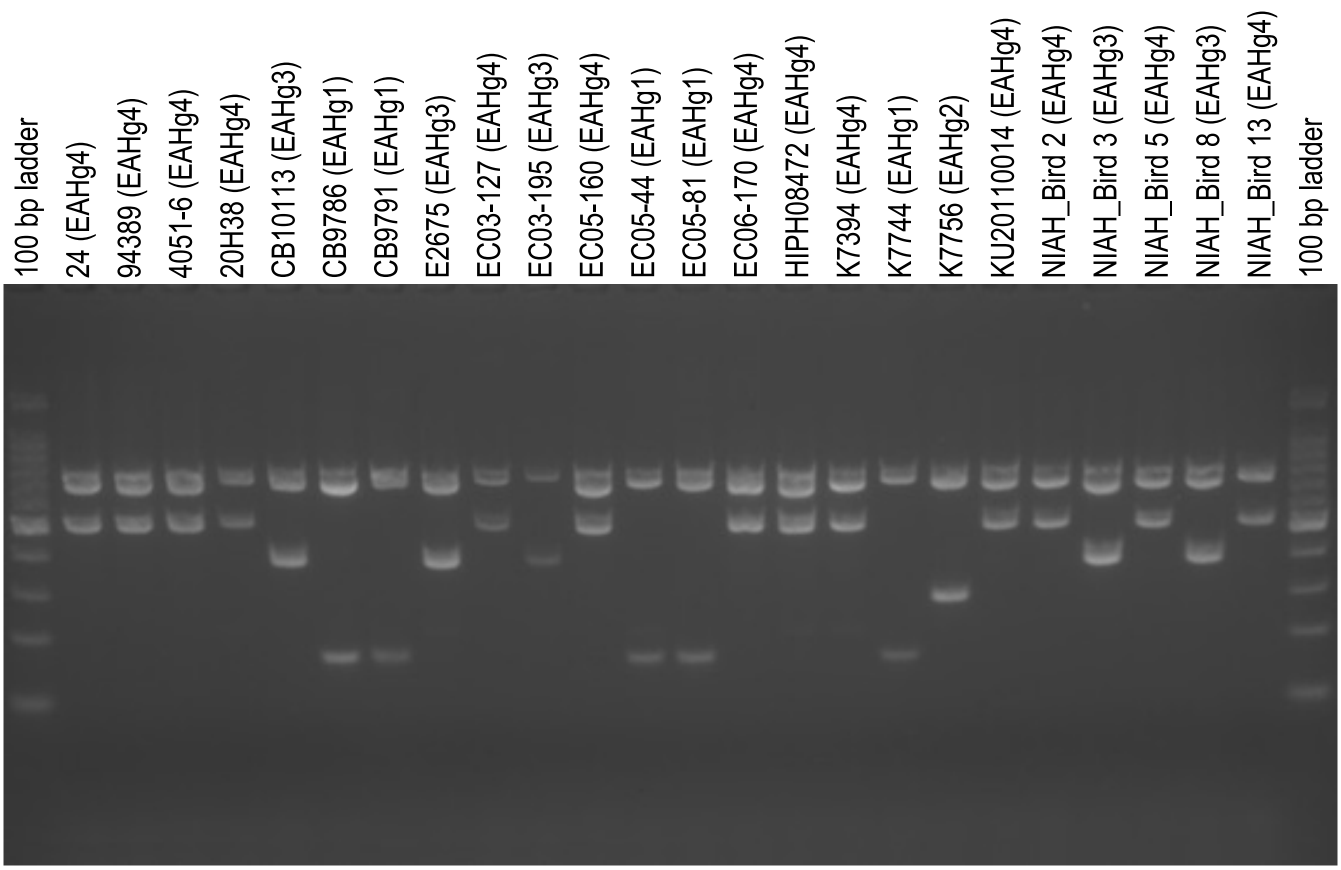

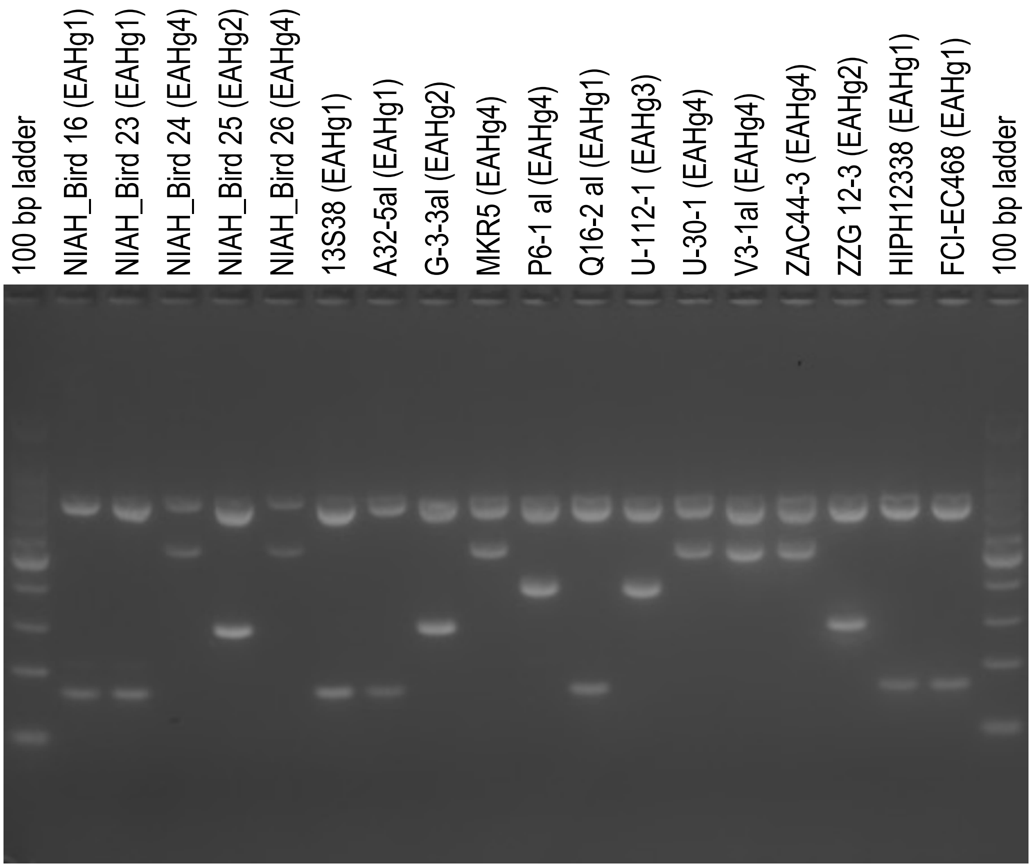

Supplementary Figure 3. Validation of EAH-genotyping PCR for the 42 strains used for *in silico* analysis.

Supplementary Figure 4

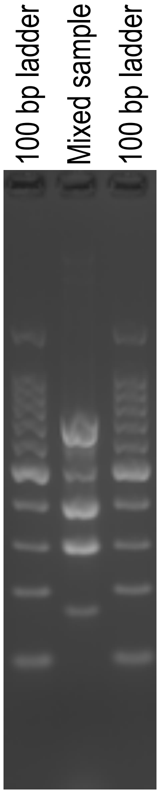

Supplementary Figure 4. Validation of EAH-genotyping PCR for the mixed sample containing strains EC05-81 (EAHg1), NIAH_Bird 25 (EAHg2), NIAH_Bird 3 (EAHg3), and NIAH_Bird 5 (EAHg4).
